# The small protein floodgates are opening; now the functional analysis begins

**DOI:** 10.1186/s12915-014-0096-y

**Published:** 2014-12-05

**Authors:** Kumaran S Ramamurthi, Gisela Storz

**Affiliations:** Laboratory of Molecular Biology, National Cancer Institute, Bethesda, MD 20892-5132 USA; Cell Biology and Metabolism Program, Eunice Kennedy Shriver National Institute of Child Health and Human Development, Bethesda, MD 20892-5430 USA

## Abstract

Aside from a few serendipitous discoveries, small proteins of less than 50 amino acids in bacteria and 100 amino acids in eukaryotes were largely ignored due to challenges in their genetic and biochemical detection. However, with the ever-increasing availability of completed genome sequences and deep sequencing, which allows analysis of genome-wide ribosome occupancy, hundreds of small proteins are now being identified. This brings to the forefront the challenges and opportunities associated with the characterization of these proteins.

See research article: http://www.biomedcentral.com/1471-2164/15/946.

## Commentary

‘Small proteins’ is a description given to proteins that traditionally escaped detection and thus detailed study due to their extremely small size. We also define ‘small proteins’ to be polypeptides that, in contrast to ‘peptides’, are encoded by small open reading frames (ORFs), are synthesized by ribosomes, and are not produced by proteolytic cleavage of a much larger precursor protein. Small proteins are difficult to identify for a variety of reasons. From a bioinformatics perspective, due to the problem of a high background, only ORFs of greater than approximately 50 or 100 codons were typically annotated as encoding proteins in sequenced bacterial and eukaryotic genomes, respectively. The lack of annotation coupled with few known phenotypes associated with mutations in small protein genes has restricted the detection of these genes by genetic approaches. Detection of small proteins biochemically requires optimized approaches so that, for instance, small proteins are not simply run off gels during electrophoresis. However, several recent lines of evidence suggest that small proteins are far more prevalent than previously imagined, indicating that a significant portion of the proteome of all organisms remains to be identified and studied.

## Increased appreciation for small proteins

In the past decade, a handful of small proteins, identified mostly by serendipity, were characterized in some detail (reviewed in [1]), and have provided interesting insights into the cellular pathways in which they participate. However, the prevalence and ubiquity of small proteins is not known. This is beginning to change. This year, several groups have made directed efforts at compiling more comprehensive lists of small proteins produced by a range of organisms. For example, Ruiz-Orera *et al*. [2] examined transcriptome data from yeast, *Arabidopsis*, flies, zebrafish, mice and humans, focusing on transcripts longer than 200 nucleotides, and identified almost 2,500 transcripts that arose from unannotated regions of the genome. Surprisingly, they found that almost all of these transcripts harboured at least one ORF with the potential of encoding a small protein of least 24 amino acids long, with an average length of 43 to 68 amino acids. Analysis of data from genome-wide analysis of ribosome-protected RNA fragments (ribosome profiling) suggested that many of these putative ORFs are translation-competent. Similarly, Bazzini *et al*. [3] used ribosome profiling in combination with computational prediction of small ORFs relying on codon conservation patterns to identify nearly 500 zebrafish transcripts that encode proteins of less than 100 amino acids in length.

While ribosome profiling has become a powerful tool to identify potential coding RNAs, a newly developed technique denoted Poly-Ribo-Seq was found to reduce the number of false positive identifications of noncoding RNAs that happen to bind ribosomes but do not get translated. This method, which exploits the property of actively translated RNAs to bind multiple ribosomes, was recently employed by Aspden *et al*. [4] with *Drosophila* S2 cells to identify close to 3,000 small ORFs likely to be translated, found either in RNA previously thought to be noncoding or in regions of RNA upstream of known ORFs. Regardless of specific numbers, it is becoming more and more clear that small proteins do not represent a fringe population of proteins and that cells may have hundreds of small proteins arising from the translation of small ORFs.

## Identifying the functions of small proteins

The identification of so many new proteins leads to the question of what they are doing. One large-scale functional study of small proteins was recently carried out by Ericson *et al*. [5], who analyzed a transcriptome study performed in *Trypanosoma brucei* and identified almost 1,000 RNA transcripts that were not associated with annotated ORFs. The authors then searched these sequences for ORFs that were at least 25 amino acids long and identified 173 small ORFs that were conserved in at least one additional member of the Kinetoplastida class. Of these, 13 small ORFs were conserved more broadly in a set of representative eukaryotes, and 63 were shown to produce a small protein product by mass spectrometry. Excitingly, RNA interference studies to knock down the functions of these genes revealed that seven of the small proteins were essential for viability. In addition, cytological studies of the proteins revealed cytosolic, mitochondrial, nuclear, and cell surface localizations. These experiments provide the first steps towards assessing cellular function for proteins whose corresponding ORFs had not been annotated previously.

The individual characterization of small proteins in bacterial as well as eukaryotic cells has also begun to reveal insights into their physiological roles (reviewed in [1]). It is striking that the majority of the small proteins that have been studied in more detail are localized to the membrane where they are required for or modulate the activity of larger membrane protein complexes. Thus, for example, Magny *et al*. [6] reported that the 28 amino acid and 29 amino acid proteins encoded by the *sarcolamban* locus of *Drosophila* regulate calcium uptake by cardiac muscles by associating with sarco-endoplasmic reticulum Ca^2+^ adenosine triphosphatase (SERCA). In *Escherichia coli*, VanOrsdel *et al*. [7] showed the 37 amino acid CydX protein associates with the inner membrane CydAB cytochrome oxidase and is required for the activity of the enzyme, and Hobbs *et al*. [8] found that the 49 amino acid AcrZ protein binds to the inner membrane component of the AcrA-AcrB-TolC efflux pump and thereby affects susceptibility to specific classes of antibiotics.

## Challenges and opportunities associated with the study of small proteins

The same features that are barriers for the identification of small proteins, such as limited sequence information, are challenges to their characterization. However, as indicated by the examples listed above, small proteins have already been shown to have important functions in the cell. In addition, experiments that overcome the experimental limitations and embrace small proteins can pay dividends for the characterization of the larger protein complexes that are targets of these proteins. For example, the inclusion of the AcrZ protein recently facilitated the structural determination of the full AcrA-AcrB-TolC efflux pump [9].

In another recent study, Allen *et al*. [10] compared bioinformatics approaches to examine the distribution of the CydX protein across bacterial species. By having a robust phenotype associated with the lack of CydX in *E. coli*, these investigators could test their predictions and the effectiveness of different identification methods. Allen *et al*. made the interesting observation that, while all orthologs are predicted to have a transmembrane domain, very few residues are conserved despite the conserved function. This is consistent with the conclusions from the previous mutational analysis that only a limited number of residues are critical for activity [7]. In addition, these investigators found that the presence of a *cydX* gene is correlated with the presence of a longer Q-loop domain in the CydA protein. We predict that further studies of the plasticity of the CydX protein and its predicted interaction with the Q-loop of the CydA protein will give insights into the activity of the important cytochrome oxidase enzymes and the evolution of protein-protein interactions.

The analysis of genes associated with the *cydAB* genes also led to the detection of two new small protein families denoted CydY and CydZ. Given that multiple small proteins also target other transmembrane proteins such as *Drosophila* SERCA, we suggest that other large membrane proteins will be subject to regulation by families of small proteins. Again, further characterization of the interactions between the different small proteins and the large protein undoubtedly will illuminate features of the protein complex. It is also exciting to think about the possibility of exploiting knowledge of the small protein families to generate synthetic peptides that have predicted and desired effects on larger proteins. Together these recent studies hint at the exciting developments that can come from surmounting the barriers that previously held back the identification and study of small proteins.
